# UK midwives delivering physical activity advice; what are the challenges and possible solutions?

**DOI:** 10.3389/fspor.2024.1369534

**Published:** 2024-06-03

**Authors:** Marina Mitra, Katherine Marino, Dane Vishnubala, Andy Pringle, Camilla Nykjaer

**Affiliations:** ^1^ Faculty of Biological Sciences, School of Biomedical Sciences, University of Leeds, Leeds, United Kingdom; ^2^ School of Medicine, Keele University, Keele, United Kingdom; ^3^ School of Public Health, Imperial College London, London, United Kingdom; ^4^ Clinical Exercise and Rehabilitation Research Centre, School of Sport and Exercise Science, University of Derby, Derby, United Kingdom

**Keywords:** physical activity, exercise, pregnancy, postpartum, midwives, knowledge, awareness, advice

## Abstract

**Background:**

Despite physical activity (PA) providing specific health benefits during pregnancy and the postpartum period, many women report decreased PA during this time. Provision of PA advice has been found to be lacking amongst midwives due to a range of barriers. This study aimed to evaluate United Kingdom's midwives' current role and knowledge regarding the provision of PA advice to pregnant and postpartum women and identify the barriers and potential solutions.

**Methods:**

Ten UK midwives (mean work experience ± SD: 15.5 years ± 10.2) participated in semi-structured interviews between May and July 2023. Data were analysed using a deductive thematic approach following Braun and Clarke's six steps. Demographic data were collected by Microsoft Forms then summarised using Microsoft Excel.

**Results:**

Six themes with 25 subthemes were identified as barriers and solutions in delivering PA advice. The role of midwives in providing PA advice during pregnancy; the role of midwives in providing PA advice postpartum; intrinsic barriers that limit PA advice provision (confidence, safety concerns, knowledge, and midwife's personal body habitus); extrinsic barriers that limit PA advice provision (lack of time, education, PA not a priority in care); solutions to allow midwives to promote PA (including formal PA education, and dissemination of resources); and optimising delivery of PA advice (personalized approach, interprofessional collaboration, and linking to mental health benefits).

**Discussion:**

Midwives consider themselves ideally placed to provide PA advice to pregnant women, with many aware of the benefits PA provides. Despite this, there is a lack of PA advice provision and knowledge of PA guidelines. Postpartum PA advice appeared to be considered outside the remit of midwives, due to limited contact. Further research is needed to determine the current level of PA advice provision for pregnant and postpartum women and explore the role of other healthcare professionals involved in maternity care.

## 1 Introduction

Physical activity (PA) is well known to improve an individual's physical and mental wellbeing (1, 2). However, PA in both the antenatal and postpartum period provides specific benefits to women. In pregnancy, this includes reduced risk of gestational diabetes (GDM) and excessive gestational weight gain (GWG) (3). PA may also reduce the risk of needing a caesarean section, particularly in obese women (4). Following birth, PA has been found to reduce the risk of postpartum depression, prevalent in 15%–20% of women; and reduce postpartum weight retention, a predictor of obesity in later life (3, 5–8).

Since 2017, the United Kingdom (UK) Chief Medical Officer (CMO) has recommended pregnant women (with no contraindications to PA) aim for at least 150 min of moderate intensity activity every week to reap health benefits such as weight control; prevention of gestational hypertension and GDM; and improved sleep and mood (9). The guidelines also recommend performing muscle strengthening activities twice weekly, as well as safety messages such as “don't bump the bump”. Guidance was updated in 2019 to include postpartum women for the first time, defined as birth to 12 months post-partum (10). Postpartum women are also recommended to aim for at least 150 min of moderate intensity activity every week for benefits including improved mental wellbeing, weight control, and improved sleep. The postpartum guidelines also recommend pelvic floor exercises, though a gradual approach to all activities is advised. For both groups, the message “no evidence of harm” is promoted. The messages in the CMO guidelines for both pregnant and postpartum women are echoed by the American College of Obstetricians and Gynaecologists and the World Health Organisation (11, 12). To ease dissemination, the CMO PA guidelines are illustrated in infographic form to be used by health care professionals (HCPs), which detail the benefits of PA and provide the key recommendations (10). Despite the benefits of PA and reassurances regarding safety, many pregnant and postpartum women do not meet the PA recommendations (13–15). Barriers to PA faced by both groups include fatigue and lack of time, confidence, and motivation (7, 16, 17).

Importantly, pregnancy is considered a teachable moment and a point where women have greater motivation to improve health behaviours such as PA and diet (18, 19). Midwives are encouraged to discuss PA at the first antenatal appointment and later on in the pregnancy if appropriate (20). During pregnancy in the UK a woman will receive seven to ten antenatal appointments with a midwife (20). This high level of contact places midwives in an opportune position to promote healthy lifestyle changes. Even brief consultations have been found to positively impact behaviour change (21, 22). Midwives therefore could play a significant role in PA promotion during pregnancy. Despite midwives being ideally placed to deliver PA advice to pregnant women, it appears this is not being provided efficiently and consistently (23). Many women have reported a lack of advice and support, with another more recent qualitative evidence synthesis of UK practice recommendations reporting that women find advice relating to weight management inconsistent and confusing (24). The postpartum period is also considered an opportunity to facilitate health behaviour change (25), but the role of a midwife is less clear, as a midwife will only typically see a woman up to ten days post-partum (26).

Many studies, both before and after the 2019 update of the CMO PA guidelines, have examined the reasons behind the lack of PA advice given to pregnant women in the UK. HCPs reported barriers include time constraints; lack of training, knowledge, confidence, and resources; and a fear of offending patients (27–30). A lack of midwife awareness of the CMO guidelines for both pregnant and postpartum women has also been identified (31). To the authors' knowledge, there has been limited research examining midwife provision of PA advice to postpartum women specifically in the UK, perhaps reflecting the delayed inclusion of postpartum women to UK PA guidelines. However, data collected prior to the 2019 CMO update identified a lack of time, training, and priority given to PA as barriers midwives face when providing postpartum PA advice (32).

It is important to hold dialogue with midwives specifically regarding the barriers they experience when providing PA advice to allow shaping of realistic solutions to support their efforts to promote PA. This study aimed to evaluate UK midwives' current role and knowledge regarding the provision of PA advice to pregnant and postpartum women, in particular the role midwives play in providing PA advice in the postpartum period due to the lack of current research. It also aimed to understand the challenges and barriers midwives face when providing PA advice and then find possible solutions to allow effective promotion of PA for both groups.

## 2 Materials and methods

### 2.1 Study design

A qualitative research study was conducted examining midwives' knowledge and delivery of PA advice for pregnant and postpartum women. This study forms part of a larger project examining knowledge and promotion of PA guidelines amongst other HCPs (33, 34). Ethical approval was given by the Faculty of Biological Sciences at the University of Leeds (27 July 2020/BIOSCI 19-039).

### 2.2 Participants

Ten UK based midwives were recruited and interviewed, a similar sample size to other related studies employing the semi-structured interview approach (28). Sample size was driven by data saturation, the point where further interviews provided no new themes (35). The inclusion criteria were midwives who trained and currently practice within the UK, able to communicate in English.

Participants were recruited by purposive volunteer sampling, primarily shared through midwife contacts and channels. The Royal College of Midwives were also contacted. Recruitment took place from April to July 2023. A brief written overview of the project was shared through these channels, alongside a Microsoft Form, where prospective participants registered interest to be contacted. The participation form collected email addresses and forms were screened to check that participants matched the inclusion criteria. After registering interest, eligible participants were emailed a Participant Information Form to keep in their personal records, and a Participant Consent Form to be signed and returned prior to demographic data collection and interview.

### 2.3 Data collection: participant characteristics

After written consent was obtained, participants were invited to be interviewed and to also complete a demographic data form. Demographic information was collected via Microsoft Forms, then automatically exported into a Microsoft Excel spreadsheet. The data were then anonymised by assigning participant unique identification numbers (chronologically by interview order). The participants were asked their gender (male, female, other), location of work (by UK region), midwifery work experience (years), current band, previous healthcare roles, and current setting of work (primary care, secondary care, combination, or other). Participants also selected one of three statements to describe their current personal PA levels: “Currently meeting the CMO PA guidelines of 150 min moderate/75 min vigorous weekly PA or combination of both”; “Currently doing some PA: >30 min moderate PA per week, but not meeting CMO PA guidelines of 150 min moderate/75 min vigorous weekly PA or combination of both”; and “Currently doing less than 30 min moderate PA per week”. Participants were not asked about strength training. The demographic data form also allowed participants to input suitable interview time, which reduced email traffic.

### 2.4 Data collection: midwife provision of PA advice

Data were collected by semi-structured interview between May and July 2023. The semi-structured approach provided a flexible approach to allow collection of rich and detailed data reflecting the midwives' individual experiences (36). The interview guide was adapted from the study by Vishnubala et al. (33) which examined doctors' attitudes towards PA. Interviews were conducted online through Zoom and Microsoft Teams depending on participant preference, to increase accessibility and convenience, and allow automatic transcription.

The interviews began with an outline of the ethical information, including participant rights right to withdraw, and anonymity. The interviews then followed four stages: demographics, PA education, PA resources and interventions, and the impact of COVID-19 on providing PA guidance. Data from the COVID-19 section was not included in analysis as it was not relevant to the research questions. The interview guide can be found in Supplementary Material.

With consent, the interviews were audio-recorded and automatically transcribed verbatim the same day by MM using recording and transcription tools on the chosen platform. The audio recordings and transcripts were anonymised and stored securely on the University of Leeds OneDrive. Prior to analysis, each transcript was checked for accurate transcription by listening to the audio recordings and any errors were corrected.

### 2.5 Data analysis

**Figure 1:**
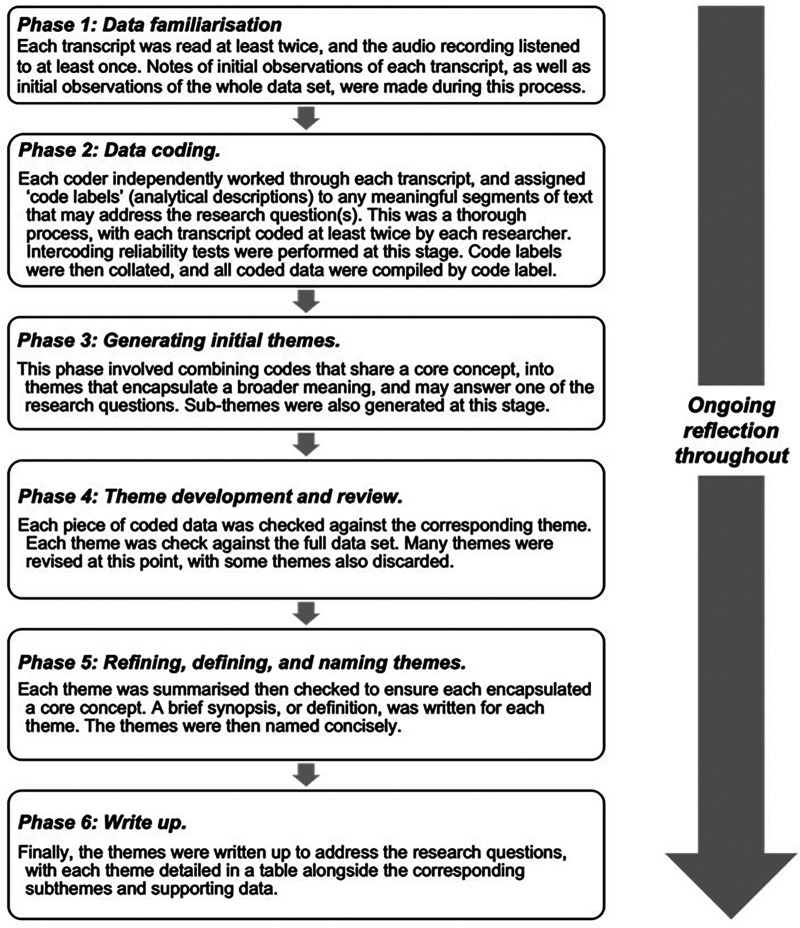
Thematic analysis methodology according to Braun and Clarke’s six steps (35).

Microsoft Excel was used to summarise demographic data. For the interview data, a six-phase deductive thematic analysis was conducted according to Braun and Clarke (35), with a flexible approach to allow critical reflection as illustrated in Figure 1. To enhance understanding, the coding process was undertaken by two researchers independently, one of whom had previous experience in thematic analysis. After each researcher coded all the transcripts, inter-coding reliability was assessed. This was calculated by dividing the number of agreed codes by the total number of codes, to allow a quick assessment, and revealed a 65% similarity (37). However, on further discussion and review of the codes, this resulted in a consensus of 75%. This is above the 70% threshold that is deemed acceptable (38). While intercoder reliability, and its appropriateness within qualitative data analysis is in question (37), the second coder allowed for richer data interpretation with greater breadth and depth of codes, that may not have been achieved with a single coder (39). A total of 203 codes were identified by the end of the coding process. During the theme generation phase, six themes and 35 subthemes were generated. This fell to six themes and 25 subthemes after themes were developed and reviewed. A reflective diary was kept throughout the process to bring awareness to the effects of the researcher's personal attitudes.

## 3 Results

### 3.1 Demographic data

Fifteen midwives completed the Initial Participation Form. Of those, nine returned the consent form and were subsequently interviewed. One additional participant was recruited directly by word of mouth, without completing the initial participation form. In total ten midwives were therefore interviewed. Sample size was driven by data saturation, the point where further interviews provided no new themes, which occurred after the ninth interview (35). Interviews varied in length between 29 and 60 min, with a mean time of 41 min.

Table 1 summarises participant characteristics. All participants were female midwives working within the Yorkshire and the Humber region. Midwifery work experience ranged from 1 to 35 years (median ± IQR: 14.5 years ± 16.25). Half of all midwives were band 6, and the most common setting of work was community midwifery. In terms of personal levels of PA, there was an equal split between midwives who met the CMO PA guidelines, and midwives who were doing some PA but not meeting guidelines. No midwives were classed as inactive (performing less than 30 min of moderate PA per week).

**Table 1:** Participant characteristics (*n* = 10).

Characteristic	Category	*n* (%)
Gender	Female	10 (100)
UK region	Yorkshire and the Humber	10 (100)
Midwifery work experience (years)	0–5	1 (10)
	6–10	3 (30)
	11–15	2 (20)
	16–20	1 (10)
	21+	3 (30)
Setting of work	Community	4 (40)
	Secondary	3 (30)
	Combination	1 (10)
	Leadership	1 (10)
	Corporate	1 (10)
Band	5	1 (10)
	6	5 (50)
	7	4 (40)
Personal level of PA	Currently meeting the CMO PA guidelines of 150 min moderate/75 min vigorous weekly PA or combination of both	5 (50)
	Currently doing some PA: >30 min moderate PA per week, but not meeting CMO PA guidelines of 150 min moderate/75 min vigorous weekly PA or combination of both	5 (50)
	Currently doing less than 30 min moderate PA per week	0 (0)

### 3.2 Themes and subthemes

Six themes were identified following thematic analysis: the role of midwives in providing PA advice antenatally; the role of midwives in providing postpartum PA advice; intrinsic barriers that limit provision of PA advice; extrinsic barriers that limit provision of PA advice; solutions to allow midwives to promote PA; and optimising delivery of PA advice. Twenty-five subthemes were also identified and listed next to their respective theme. Sub-themes were discussed with CN and confirmed with both CN and KM.

#### 3.2.1 Theme 1: the role of midwives in providing PA advice antenatally

Table 2 summarises the role of midwives in providing PA advice antenatally, including five subthemes. Square brackets following each quote indicate the participant's unique identifying number. Many midwives expressed that pregnancy was an opportune time to share PA advice with an appreciation of its importance and the benefits. However, many midwives noted an under-provision of PA advice during pregnancy, with discussions lacking in regularity, length and depth. There was a variable awareness of resources regarding PA in pregnancy. Few midwives were aware of the CMO PA guideline infographics, and some midwives were not aware of any resources related to PA in pregnancy. There was also a focus on providing PA advice to women with raised BMIs (Body Mass Index).

**Table 2:** Subthemes for “the role of midwives in providing PA advice antenatally”.

Subtheme	Example quotes
Midwives are ideally placed to provide PA guidance during pregnancy	“*I feel that you're in a really advantageous role where you can actually promote health and wellbeing, and that's part of it. It's like diet, exercise, smoking, drinking. It's like that unique opportunity. Everyone accesses a midwife, so you have got, you’re in that position to be able to deliver that*” [008] “*A lot of people want to kind of be a bit healthier once they’re pregnant because it's not just prioritizing themself anymore, they're kind of thinking about the baby and themselves as a mum… And so, I do think kind of just that point of coming from a health professional, at a time when you really do wanna sort of like look after yourself, is gonna be a bit more poignant*” [001]
Midwives appreciate the importance of PA in pregnancy	“*I think it's something that's valuable for pregnant women, and it's something that we should be promoting to everybody as long as it's safe for them to do so.*” [003] “*It's really important. Really setting this this lady up for the transition through pregnancy and postpartum.*” [007] “*Actually, you know the evidence is, you know, that the more active in pregnancy somebody is, the better outcomes are, and for, and their mental health and well-being both of, and labor, birth outcomes as well*” [002] “*It's a basic thing that can have such health benefits and have health benefits for pregnancy… it's sad that we see a lot of women with yeah, one, raised BMI, two, with gestational diabetes, and three with mental health problems. And physical activity can have a benefit towards all those things.*” [005]
The lack of discussion of PA in pregnancy	“*As a sort of general advice, I would still recommend to women that they incorporate exercise into their daily routine. But it's not something that I would regularly give advice on*” [003] “*It's just such a brief conversation.*” [001] “*I don't think we do talk about it enough at booking on reflection*” [005] “*It was always kind of pushed off in that, oh do you exercise? Oh well just you can just keep doing what you're doing and it wasn't really any further than that in terms of depth*” [007]
Variable awareness of resources of PA in pregnancy	“*I'd usually refer to something like the Royal College of Midwives Resources, or the Royal College of Obstetrics and Gynae*” [003] “*I don't know of any like particular guidelines*” [001] “*I usually refer to the guidance from the Chief Medical Officer that this really good infographics that I usually try and share, and it talks about the recommended exercising in pregnancy.*” [002]
There is a focus on women with raised BMI	“*I think I speak more to women who've got a raised BMI at booking about doing a bit of regular exercise and how can have positive impact on them developing things like, because they are slightly higher risk of getting things like raised blood pressure and pregnancy, and diabetes in pregnancy and just try to reduce the chance of becoming, having hypertension or diabetes.*” [005] “*I'd say the women who have a raised BMI are definitely people that I would spend a little bit longer discussing this because this comes into our health and well-being and our public health*” [007]

#### 3.2.2 Theme 2: the role of midwives in providing postpartum PA advice

The role of midwives in providing postpartum PA advice was also explored (Table 3), and four subthemes were found. There was a variable level of postpartum PA guidance, and many midwives noted that postpartum PA discussions focused mainly, or occasionally solely, on pelvic floor exercises. Postpartum PA advice was often felt not to be “appropriate”, due to the recentness of birth. PA discussions also highly depended on a woman's mode of delivery. Many reported that post Caesarean section, women receive more information and physiotherapy support, support not provided to women post vaginal delivery.

**Table 3:** Subthemes for “the role of midwives in providing postpartum PA advice”.

Subtheme	Example quotes
Variable level of postpartum PA guidance	“*I feel sort of like that postnatally I don't give as much advice, because they're not with us for quite as long postnatally. And in that initial period that I'm supporting them, we wouldn't necessarily be suggesting very much in terms of exercise*” [002] “*It wasn't part of the discharge bundle, so we just didn't discuss it*” [006] “*I would make sure that I discussed it with everybody, as just sort of part of discharge home from hospital, and ongoing care of themselves.*” [003] “*It's kind of not really in our remit.*” [006]
Focus on pelvic floor exercises	“*We mainly focus on pelvic floor exercises as a discharge chat.*” [004] “*What's interesting is a lot of people bring in pelvic floor at that point so as much as pelvic floor exercises are an activity it's more, my focus was more on physical, and the pelvic floor would come after. I think maybe sometimes there needs to be a little bit of clarification of, of separating those 2 things.*” [007]
Postpartum PA advice is not always appropriate	“*I think it is just like how recently they've had a baby that it's just not appropriate.*” [001] “*It doesn't feel like a great opportunity to talk about this*” [002] “*I might not even mention it because it just depends on what level they're at as to whether they're gonna be open to that or not, whether they’re on day three or just come out of hospital, or whether you know it's been discharged, it's it. It's not always appropriate to deliver at that time.*” [008]
PA guidance depends on mode of delivery	“*And then postnatally it's, it's very much tailored the advice I give around mode of birth. So my advice is very different to somebody who's had a normal vaginal delivery, to someone who's had a caesarean section*” [007] “*And so I think there those women who have had a section probably do, are told a bit more about physical exercise than perhaps those which haven't, just because they haven't had someone specifically talk to them about that.*” [010] “*Women who have had a Caesarean section are given a very basic leaflet about exercises to do after they've had a section. But I don't think they get any sort of leaflet around exercises to do postnatally if they've had a normal birth*” [005]

#### 3.2.3 Theme 3: intrinsic barriers that limit provision of PA advice

There were three subthemes regarding intrinsic barriers limiting provision of PA advice (Table 4). Confidence levels varied between midwives, but also depended on the current level of PA of the woman. Many midwives also cited safety concerns as limiting their confidence to provide PA advice, particularly encouraging the adoption of new PA. Lack of knowledge and understanding of PA guidelines were common themes throughout, with low awareness of resources such as “Moving Medicine”. A midwife's personal body habitus was also discussed as a potential barrier, with both midwives of higher and lower BMIs finding it impacted provision or reception of PA advice.

**Table 4:** Subthemes for “intrinsic barriers that limit provision of PA advice”.

Subtheme	Example quotes
Lack of confidence	“*I think my confidence levels are probably quite low because it's not really something I feel I know a lot about.*” [003] “*I feel confident in delivering it to somebody who is already active.*” “*I'm not so confident with somebody who is quite inexperienced with exercise at all*” [007] “*So obviously we've got to like make sure they're safe. It's probably just like better not to say that much*” [001]
Lack of knowledge	“*I don't know of any like particular guidelines*” [001] “*It's not really something I feel I know a lot about*” [003] “*Lack of knowledge. Yeah. Lack of training, lack of understanding*” [007]
The effect of midwife personal body habitus	“*And I do know, because I've spoken to them, some women, some midwives, who don't do much exercise and have put on a lot of weight themselves say I find it hard to give that sort of advice because I'm not being a very good example to women*” [005] “*Because I'm quite petite myself, I'd have to be careful that wasn't intimidating the patient who maybe had a raised BMI. Because you know I've had comments made you know you're not pregnant and you've probably got quick metabolism, and I look at cookie and put on 5 pounds*” [007]

#### 3.2.4 Theme 4: extrinsic barriers that limit provision of PA advice

There were four themes regarding the extrinsic barriers limiting PA advice provision (Table 5). Lack of time was reported by all midwives as limiting provision of PA advice. Many midwives highlighted the sheer volume of general pregnancy information to be communicated in appointments. Lack of midwifery education was also a major theme, at both undergraduate and postgraduate level. Most midwives had little to no experience of PA education as a student midwife, with PA covered as a topic within a lecture, if at all. Concerning postgraduate training, no midwives had received PA training within mandatory training or study days. Many midwives instead relied on self-directed learning regarding PA in their own time. PA not being seen as a priority was also commonly noted. A woman's personal attitude to PA was discussed to also act as a barrier, with midwives finding discussing PA can be a difficult or touchy situation, with concerns that women may feel judged or patronized.

**Table 5:** Subthemes for “extrinsic barriers that limit provision of PA advice”.

Subtheme	Example quotes
Lack of time	“*I think certainly, for one of the things that I find hard, is the time that it feels like this, less time to do more things and sort of give more information and evidence that we've given information and that sort of feels so hard to do*” [002] “*There's not that much time antenatally there's not, probably even less postnatally*” [008] “*I make time. I don't care if I run over.*” [009]
Lack of education	“*We didn't receive a lot of training about physical exercise. I don't think it's really something they really talk about a lot when you're doing it, when you're a student midwife. I don't recall ever having really a session on promoting physical exercising in women*” [010] “*Pretty much zero, I would say. Um, it's only something I've looked and researched myself and found out*” [008] “*We don't really have a lot of up to date information. It's not really something that is incorporated into our mandatory training that we do*” [003] “*It is very much dependent on that individual midwife to go out and find the research themselves.*” [007]
PA is not seen as a priority	“*It's not a big topic within midwifery*” [001] “*There's other topics that probably always gonna take precedent.*” [002] “*It can easily get forgotten about because there are so many other things we're having to focus on and make sure that the baby's well. I think sometimes physical exercise just get sort of pushed to the side a little bit, forgotten about, which you know is understandable because it's not always a priority*” [010]
Women may not always welcome PA discussions	“*It's a difficult conversation to have. They're already on the defensive so you've got to just be mindful, about how, I mean, it's an appropriate conversation*” [006] “*It can be a bit of a touchy subject*” [010]

#### 3.2.5 Theme 5: solutions to allow midwives to promote PA

The participants proposed several solutions to enable midwives to promote PA, which emerged as five subthemes (Table 6). Many midwives suggested inclusion of PA in midwife training and assessment, at both undergraduate and postgraduate level, such as incorporating PA into mandatory training. A second subtheme that was identified was the need for PA to be considered with increased priority. Many midwives stressed the need for improved dissemination of resources on PA, including utilising online platforms such as the trust intranet, or QR codes. Ensuring access to local services, (such as pregnancy-specific classes, free or subsidised gym schemes, or hospital-run classes) was also highlighted, with midwives wishing for free and safe services to recommend. Other opportunities for sharing advice with women were also suggested, including parent education sessions, television campaigns, and display of resources in waiting areas. Despite the interviewer asking about solutions regarding provision of PA to both pregnant and postpartum women, most participants discussed solutions mainly in the context of antenatal advice.

**Table 6:** Subthemes for “solutions to allow midwives to promote PA”.

Subtheme	Example quotes
Inclusion in formal training	“*If student midwives are having a lecture on gestational diabetes, maybe incorporating how physical activity can really support with gestational diabetes symptoms*” [003] “*Think you would need to actually do a proper session. And then get it incorporated into their standards that they need to achieve as something that needs to be signed off*” [006] “*An OSCE scenario, or a viva, or giving the student a scenario about a 24 week pregnant lady who's had her first baby she's in an antenatal appointment, about physical activity in pregnancy? What would your advice be? And that OSCE would be an amazing place to start, and putting it as important as the other conversations that we have*” [007] “*I think it should be as part of our mandatory training when I think about it now.*” [007]
PA needs to be considered a priority	“*I think seen as a priority and you know, because we talk about certain foods to avoid, to stop them getting food poisoning but actually the risk of them getting salmonella and listeria and toxoplasmosis is so much less compared to the likelihood of them getting high blood pressure and diabetes because they're not exercising*” [005] “*Encouraging physical activity in pregnancy needs to be at the top of the agenda. If we look at the risk factors, if we look at the growing rates of mental health illness in pregnancy and outside pregnancy but also the risks postpartum of then developing more serious mental health illness. And then if we look at the rising rates of obesity, diabetes, DVT*” [007]
Dissemination of resources to midwives	“*I think there needs to be a hub online. That we click on the hyperlink and it takes us straight through to everything we need to know*” [006] “*So they should be as part of our trust guidelines so within our policies and protocols that would be really good to put it there. And then with links to where it's published.*” [007] “*I think emailing them around to the midwives or putting them, we have, you know, on a staff internet somewhere there, maybe making a little guideline about physical exercise.*” [010] “*It's quite nice to sort of be 3 clicks away from the thing that you want to show them*” [006]
Ensuring access to local services	“*But I do think just having a class that we were safe to sort of recommend would be a key one*”*.* [001] “*I think pregnant women maybe need to have access to 20 h of exercise at their local sports centre. And, because actually if women are offered something they will take it*” [006]
Opportunities to share PA advice with women	“*Doing like an advertisement on the telly that everybody sees and watches that, you know, it's okay to do this*” [008] “*I think if we brought it into our parent education sessions it would be really helpful*” [007] “*Use social media, but make sure that they put up in all clinics, you know in in like antenatal/ postnatal clinics*” [009]

#### 3.2.6 Theme 6: optimising delivery of PA advice

Four subthemes emerged regarding methods by which individual midwives may optimise delivery of PA advice (Table 7). Many midwives discussed how a personalised approach helps PA promotion. Midwives also suggested PA could be discussed further along the pregnancy journey, with one midwife also suggesting postpartum PA advice be incorporated into antenatal care. Many midwives stated that PA advice was often included as part of mental health conversations and promoted through that. The need for inter-professional collaboration was discussed by all midwives. Midwifery support workers, physiotherapists, doctors, and exercise specialists were mentioned as professionals who could help promote PA. Again, similar to theme five, most participants discussed methods mainly in the context of delivering PA advice to pregnant women specifically.

**Table 7:** Subthemes for “optimising delivery of PA advice”.

Subtheme	Example quotes
Personalised approach	“*With any advice and pregnancy, talking to people about what they already understand and know and do, and making it a more personalized conversation. It's always gonna, it's a, people always gonna respond back to that, than just feeling like this is, this is generic advice, here's a leaflet.*” [002] “*So I think it's just been really understanding their situation, asking her how she feels about exercising, what exercise she did before, what exercise does she enjoy*” [010] “*And it's a conversation more than me just giving information as much as I can possibly do. So I think that it's that I think you know it's, yeah, it's more woman led.*” [009] “*It's a wide variety really and you kind of have to tailor your information to what the women actually need.*” [006]
More regular conversations throughout pregnancy	“*It probably needs to be something that's maybe more regularly discussed throughout pregnancy. I'm aware that community midwives would discuss it booking, but it might be something that would be a good idea to revisit throughout the pregnancy*” [003] “*I think it's something we should be exploring later, in more depth say at 16 weeks when people are starting to feel a bit more full of energy and then revisit it and then suggest things that were, that are more practical*” [009] Discussing postpartum PA advice: “*It just could be done better and, and I think that would be perhaps a little bit more information antenatally, but it's really difficult to know where that would be slotted in*” [009]
Link to mental health	“*I think like showing evidence of how it's gonna impact mental health*” [001] “*Mental health probably is another trigger for speaking about exercise*” [005] “*I would definitely make sure I did it in more depth with women with mental health issues.*” [006]
Interprofessional collaboration	“*If we had more of a collaborative approach, if we had closer links with say physios, experts in physical activity and if we had that option to be able to, to discuss individual cases, I think that would be very helpful*” [007] “*I think what would be really interesting would be to allocate a champion for each area. So if we had a obstetrician who was a champion for physical activity and then if we had a physio who then worked, and we brought together like a collaborative working group, then if we had a fitness expert and then we had say a few members from the community midwives team, a member from the antenatal clinic where they all have the physical activity at their kind of priority, then that information could be, that could be a collaborative working group*” [007] “*I think it's got to be across the board really. I think it's got to be from the doctors as well. Not just the midwives and not, and I say, from the support workers because they are often the ones who do see with a very, very first appointment is generally with a support worker*” [005]

## 4 Discussion

This study aimed to evaluate midwives’ current role and knowledge regarding the provision of PA advice to pregnant and postpartum women. It aimed to understand the challenges and barriers midwives face when providing PA advice and find possible solutions to allow effective promotion of PA for both groups. To this research team's knowledge, this was the first UK study to explore the role of midwives in providing PA advice to both pregnant and postpartum women; and examine the challenges and possible solutions regarding PA promotion for both groups. This study found midwives considered providing PA advice to pregnant women as within their role, but not to postpartum women. A clear lack of PA advice provision was identified, with barriers including a lack of confidence, knowledge, time, and education. A number of solutions were proposed to allow effective promotion of PA, and to optimise delivery.

### 4.1 Midwives' current role and knowledge

Although NICE guidelines (20) for antenatal care include providing PA advice, the role midwives play in this provision was unclear from the guidelines. Midwives are arguably ideally placed to provide PA advice in pregnancy due to the early contact and universal access to midwives for all pregnant women. Indeed, McParlin et al. (30) had similar findings when examining midwife provision of PA advice to obese women amongst a large sample of midwives (*n* = 192) practising in the North East of England, with midwives considering PA promotion part of their professional role. Pregnancy was also considered an opportune time for health promotion, when women are motivated to make healthy lifestyle choices thus supporting ideas discussed by Rockliffe et al. (18). It is encouraging that many midwives appreciated the benefits provided by PA during pregnancy, including control over conditions such as gestational diabetes and raised BMI, both of which are supported by strong evidence (3). Despite the potential benefits, PA lacked discussion in pregnancy, corroborating the findings of Brown and Avery (23), who reported that women's experiences of PA advice were lacking.

This study helped to clarify the perceptions of midwives and their role in the postpartum period, previously lacking in research. Postpartum PA advice was felt by many to not be appropriate, or outside a midwife's remit, due to recentness of delivery. Interviews revealed a large variation in the dissemination of postpartum PA advice provision, with some midwives not discussing it at all. The findings of this study echo the results of Haakstad et al. (40) where only 40% of Norwegian midwives (*n* = 65) were found to provide postpartum PA advice as opposed to 95% at the first pregnancy related visit. The discrepancy between the involvement of UK midwives in pregnancy vs. postpartum PA guidance may reflect the NICE guidelines. NICE guidelines state PA *should* be discussed antenatally (20), but PA *may* be discussed postnatally (26). However, NICE specifies pelvic floor exercises as part of the discharge conversation, which accounts for the number of midwives in this study who solely focused on pelvic floor exercises.

There was also limited knowledge of the UK CMO PA guidance, with only two out of the ten midwives aware of the pregnancy guidance, aligning with the findings of the study by Taylor et al. (31), where only 33% HCPs were aware of the CMO guidelines in pregnancy. However, no midwives in this study were aware of the postpartum CMO guidance, compared to the 30% found by Taylor et al. (31). Differences such as a much larger sample size, and involvement of HCPs besides midwives (*n* = 393, 37% midwives) may account for disparities in findings. However, in this study, the midwives aware of the CMO PA infographics found them useful, identifying a missed avenue by which midwives may improve their practice. The need for dissemination of the 2019 guidelines to key stakeholders was highlighted by Foster in a 2018 presentation, and this study's findings suggest this need still exists (41).

### 4.2 The barriers faced by midwives

Midwives faced many barriers when providing PA advice. Lack of confidence in providing information on PA was commonly discussed, impacted by factors such as safety concerns. Low confidence was also found as a barrier in the De Vivo and Mills study (28) in their interviews of ten community midwives, where lack of confidence led to PA advice being limited to basic advice. Similarly, our study found midwives provided generic advice, or held back advice due to safety concerns. Additionally, some midwives were less confident with less physically active women, a group that could be seen as a priority. Numerous studies have identified lack of knowledge as a barrier prior to the release of the 2019 CMO guideline update (29, 30), but this study suggests there has been no improvement in knowledge levels, highlighting the poor dissemination of resources. Bright et al. (27) found in their study of Welsh midwives (*n* = 1,338), that some midwives may feel hypocritical when providing PA advice, due to their own lifestyle, thoughts reflected by midwives in this study, but conversely, they may also be well placed to understand the difficulties of being physically active and have insight and empathy. However, our findings suggest that difficult conversations were not limited to midwives with higher BMIs: one midwife, who described herself as petite, found providing PA advice to women with raised BMIs was not always received well, due to a perceived lack of understanding of the woman's situation.

In terms of extrinsic barriers, lack of time was a predominant theme throughout all interviews, aligning with current research (28, 32). This reflects the wider resourcing based issues such as staffing in the NHS (42). Although midwives have a “high” contact with pregnant women, the list of topics to be covered is extensive (20), as many midwives discussed. However, in this study, two midwives stated they provided PA advice despite time pressures, but at the cost of other topics, or running over appointments. Lack of education regarding PA has been identified as a barrier in multiple studies (28, 29, 32), with this study finding many midwives experienced minimal undergraduate PA training, and almost all midwives reporting no formal postgraduate PA training. However, despite this, many midwives in this study discussed how their personal interest in PA had driven them to seek out information in their own time and this aligns with other research into HCPs and their training on PA (42). However, the failure to include PA in formal training and subsequent need for reliance on self-directed learning may add pressure to midwives who are already under stress, as many in the interviews discussed. Many midwives commented that PA was often not a priority or forgotten compared to other topics, such as the baby's growth and health, that instead took precedent. This contradicts the findings of the Bright et al. (27) study, where only 6.1% of midwives felt there were more important things to do. However, the Bright et al. study was conducted in the first half 2019, it may be that in the four years since data collection by Bright et al. (27) factors such as COVID-19 and increasing mental health problems, have pushed PA to the side in appointments (43).

### 4.3 Solutions and optimising delivery of PA

This study explored midwives' perceptions of how PA advice provision may be practically improved, a topic with limited coverage besides the De Vivo and Mills (28) study. Both undergraduate and postgraduate curriculums were noted to be lacking in PA content, providing an opportunity for improvement, as many midwives discussed. Both this study and the De Vivo and Mills (28) study found midwives suggested PA be incorporated in mandatory training and the student midwife curriculum. This study also found additional suggestions including methods for assessment, such as OSCE (Objective Structured Clinical Examination) scenarios. The idea that midwives should not be expected to complete this training in their own time, was also discussed, to encourage maximum engagement. These suggestions are supported by the findings of Taylor et al. (31), where an education programme significantly increased HCP confidence and frequency of PA conversations with both pregnant and postpartum women. The education programme, titled “This Mum Moves”, aimed to provide HCPs with education regarding the pregnancy and postpartum PA guidelines. Prior to training, Taylor et al. (31) found only 5.6% of midwives were “confident” or “highly” confident providing PA advice to pregnant women, which rose to 71.7% after training (*p* < 0.001). Similarly, the proportion “confident” or “highly” “confident” providing PA advice to postpartum women rose from 5.6% to 72.8%. Dissemination of resources was another theme that many midwives proposed as an actionable solution. Many midwives suggested utilising electronic platforms to share resources, such as an online hub, integration into trust guidelines, or inclusion in emails. Many reported their trust had recently switched to paperless notes, which some suggested that if electronic resources were utilised effectively, it may buy midwives back some time to have meaningful conversations. Also, some midwives proposed putting PA resources in a central location, so they can be obtained as quickly as possible to help patients efficiently. Additionally, seeing PA as a priority was emphasised by several participants, including one who stated that “encouraging physical activity in pregnancy needs to be at the top of the agenda”.

Methods individual midwives could take to optimise PA delivery were also suggested. Novel methods of optimising delivery of PA advice, aligned to the needs of women emerged, of which had not been seen in previous similar studies. This includes incorporating PA into mental health discussions, which is often discussed due to the high prevalence of mental health problems in both pregnancy and the postpartum period (6). As there is strong evidence for PA reducing risk of postpartum depression (3), including PA in mental health discussions with women is an appropriate time to fit postpartum PA into appointments, without it feeling like an extra topic. NICE guidance states PA should be discussed at the booking appointment, but it is at the individual midwife's discretion whether PA advice is revisited throughout pregnancy (20). However, many midwives suggested incorporating PA into discussion more regularly through pregnancy as a way of optimising delivery, especially due to a woman's fluctuating energy levels and severity of pregnancy side-effects. One midwife also suggested postpartum PA advice be incorporated into antenatal appointments. Although NICE guidelines do recommend midwives discuss the postpartum period during pregnancy regarding topics such as feeding, there is no mention of postpartum PA (20). Like Walker et al. (19) who suggested contraception, usually only discussed following birth, be incorporated antenatally to improve provision, the same could be done for PA. The need for interprofessional collaboration was discussed by many midwives, with midwifery support workers, physiotherapists, doctors, and exercise specialists mentioned as professionals who could help support provision of PA advice. This is particularly important if any pregnancy or postpartum complications arise, referring pregnant and postpartum patients to the care of qualified exercise specialists will remove the midwife's responsibility to provide comprehensive guidance on what the woman can and cannot do in terms of PA. There was a suggestion of establishing a PA clinical champion within each HCP field; similar to a solution proposed in the De Vivo and Mills (28) study, highlighting a practical approach by which interprofessional collaboration may be facilitated. Other approaches included joint clinics and multidisciplinary teams to discuss individual cases. This means midwives will be adequately supported by HCPs and exercise professionals, who may have more knowledge and experience in providing more personalised PA advice.

### 4.4 Strengths and limitations

This study has several strengths. The use of semi-structured interviews allowed for rich data collection (36). The study also identified novel findings such as the inclusion of PA advice for mental wellbeing. Furthermore, as it is important to learn from the process of conducting research and evaluations (44), this study details what worked well and how, when undertaking research with midwives, it can help to inform future investigations. A rigorous approach to data analysis was taken by following Braun and Clarke's six steps as a guide (35). Additionally, the use of two coders reduced subjectivity, and discussion of coding allowed for consensus and enhanced understanding (39). Training on data collection and refinement of interview techniques was conducted, in order to help participants share their informative accounts. Despite the small sample size, this study reached saturation, and has a similar number of participants to other related studies (28). However, as all participants were working in the Yorkshire and Humber region, findings may not reflect the whole UK, as practice may differ by trust. Although participants were from the same geographical area, there was a range in setting of work, band, and years of experience.

It is likely that midwives who are willing to be interviewed in their personal time may be more invested in providing PA advice. This volunteer bias means the views and experiences of the midwives in this study may not accurately reflect all midwives. Additionally, this sample of midwives all perform regular PA, with none classed as physically inactive. This may also influence participation and nature of responses. However, even so, these midwives who are more likely to have an interest in PA, still felt lack of knowledge, confidence, and education in PA affected their practice, themes that will likely also ring true for the general midwife population.

### 4.5 Implications for practice and research

We aim to share the findings of this research widely through various regional and national PA networks such as the Regional Public Health Networks, the Regional Physical Activity Delivery Networks (e.g., Active Leeds, and Active Partners Trust/Making our Move and Move More Derby in the East Midlands), the UK CMO Physical Activity Communication Network, and the Moving Medicine Network. The goal is to ensure that colleagues can benefit from the insights gained during both the research process and the outcomes.

To determine the current level of PA advice provided to pregnant and postpartum women, a service evaluation or clinical audit across multiple NHS trusts may be beneficial. The findings from this study could help shape the questions to be asked. This would encompass all midwives within a trust, not just those who had expressed interest and therefore more likely to provide PA advice. Future research should also include midwives with lower levels of PA themselves and allow exploration of how this may affect the advice they give, and the barriers and facilitators they encounter when doing so. In addition, just as the Taylor et al. (31) study examined the effect of postgraduate training on midwives' confidence and knowledge regarding PA, a similar study could be conducted for student midwives, to provide rationale for the inclusion of PA in UG curriculums. In this study, there was a sense that postpartum PA guidance fell outside a midwife's remit. Therefore, it would be useful to conduct similar studies on health visitors, GPs, and midwifery support workers (HCPs identified by the midwives in this study as potentially involved in providing PA advice) to determine who, if anyone, has the role of providing postpartum PA guidance.

## 5 Conclusions

This was one of the first studies to address midwifery PA advice provision to both pregnant and postpartum women. Though midwives consider themselves ideally placed to provide PA advice to pregnant women, postpartum PA was felt to be outside the remit of midwives. Many barriers such as lack of time, education, and confidence were expected based on previous studies and were found as subthemes in this study. Multiple solutions were proposed, some of which bear similarity to the suggestion of existing studies, but many solutions are novel and locally actionable. Further research is needed to determine the current level of PA advice provision for pregnant and postpartum women, the determinants inactive midwives encounter when promoting PA, and also explore the role of other HCPs involved in maternity care.

## Data Availability

The datasets presented in this article are not readily available because of concerns about participant privacy and confidentiality. Requests to access the datasets should be directed to c.nykjaer@leeds.ac.uk.
