# Supplementary material for: UK midwives delivering physical activity advice; what are the challenges and possible solutions?
Source: Front Sports Act Living. 2024 Jun 3;6:1369534. doi: 10.3389/fspor.2024.1369534 (PMC11180801; doi:10.3389/fspor.2024.1369534)
Supplement: Supplementary file 1 [file Table1.docx]

Supplementary Material

Interview guide

[Thank the participant for attending the interview]

[Participant to have read the PIS and consent form, and returned a completed copy of the consent to the researcher prior to the interview]

[Researcher and participant to introduce themselves. Researcher to introduce the evaluation and the purpose of the interview]

[Reiterate that the information participants provide will be anonymised and confidential. **Check that the participant is comfortable with the interview being recorded**]

[Start recording and transcription].

For the purposes of the recording, can I just confirm you are comfortable with this interview being recorded.

In this interview I am interested in hearing about your experiences of delivering physical activity (PA) guidance to women, your opinion as a clinical expert on the barriers, challenges and solutions to improving midwives providing PA advice and views on current interventions/developments.

Please be assured that you will remain anonymous and the research team will not share your comments with anyone else, so be as honest as you can. If there are any questions that you would prefer not to answer you do not have to answer them. If at any point you do not understand what I am asking or need some clarification, please feel free to ask as we go along. You will be given an opportunity to say anything that we have not covered at the end of the interview

Do you have any questions about the interview before we begin?

# DEMOGRAPHICS

**Q1. State your role, experience and current location of work (community or secondary care)**

Q1a. Do you work in community or secondary care?

Q1b. What is your specific role?

Q1c. How many years’ experience post-graduation do you have?

Q1d. What is your current band?

Q1e. Have you had any previous qualifications/training or healthcare roles prior to midwifery?

# PA EDUCATION AND RESOURCES

**Q2. Tell me about the education and training that you have received in relation to PA.**

[Education and training during formal previous and midwifery degrees and Continuous Professional Development;

Feelings/confidence around delivering CMO PA guidance]

**Q3. What do you currently do in terms of delivering PA guidance to pregnant women?**

[Explore who delivers guidance, whether part of standard procedure or ad hoc, whether content of guidance is general advice or adheres to guidelines

How do you decide who to provide PA advice to?

Are there specific groups that are prioritised?

Timing of advice?]

Q3a. Why this approach;

Q3b. What s/he thinks of this approach;

Q3c. What works well and why?

Q3d. What individuals/practice could do differently?

Q3e. What needs to change for this to happen?

Signposting, etc.

**Q4. What do you currently do in terms of delivering PA guidance to post-natal women?**

[Explore who delivers guidance, whether part of standard procedure or ad hoc, whether content of guidance is general advice or adheres to guidelines

How do you decide who to provide PA advice to?

Are there specific groups that are prioritised?

Timing of advice?]

Q4a. Why this approach;

Q4b. What s/he thinks of this approach;

Q4c. What works well and why?

Q4d. What individuals/practice could do differently?

Q4e. What needs to change for this to happen?

Signposting, etc.

**Q5. What would be your ‘top tip’ for promoting PA to pregnant women?**

[Explore what they do well and would share with their fellow colleagues as something that is exemplar practice]

**Q6. What would be your ‘top tip’ for promoting PA to post-natal women?**

[Explore what they do well and would share with their fellow colleagues as something that is exemplar practice]

**Q7. Are you aware of any physical activity guidelines specific to pregnant and post-natal women?**

[e.g WHO, CMO]

Q7a. How did you come across it?

Q7b. Are they useful

Q7c. Is there anything else you would find useful?

**Q8. Are you aware of the CMO PA guidelines for pregnant women?**

Q8a: If yes- how did you come across it, format, is it useful?

Q8b: where would be the best place to put/share them to increase access?

**Q9. Are you aware of the CMO PA guidelines for post-notal women?**

Q9a: If yes- how did you come across it, format, is it useful?

Q9b: where would be the best place to put them to increase access?

**Q10. What would help you to deliver PA advice to your patients?**

[Prompts here, time, resources, partnerships with providers, better training, other people I could refer to in house, policy commitment for PA promotion ]

**Q11. What in your opinion is the challenges and barriers that prevent midwives giving PA advice?**

[Prompts here-Consider individual, environment]

**Q12. How do you think we could engage hard-to-engage midwives who are less enthusiastic or even anti PA?**

Q12a. What works: why?

Q12b. What works less well and why?

**Q13. If midwives are hard to recruit to deliver this advice, are there other HCPs that are better placed to deliver PA advice to pregnant and post-natal women?**

[e.g. doctors, physiotherapists]

**Q14. What education out there for midwives do you feel is most effective at enabling midwives to promoting PA?**

[Prompts

The 2019 guidelines

Infographics

PA training

Mentoring

What works well and why?]

**Q15. How can we embed more PA into undergraduate and postgraduate curriculums?**

[Consider delivery type/method, scalability, consider assessment]

**Q16. What are the solutions to increase midwives giving PA advice?**

[Consider policy, motivating practices, clinicians]

## 2019 PA guidelines

*(Skip to Q21 if unaware)*

**Q17. Do you think the 2019 update of the CMO guidelines has been helpful or not?**

Q17a. Why is this?

**Q18. Have you seen the CMO PA guidelines in infographic form for pregnancy and post-natal women? If so which ones?**

Q18a. If yes- how do you use it?

**Q19. What else would you like to see in the guidelines?**

[Prompts: 24 hour message, inclusion of guidelines on sleep and PA, specific diseases, other groups?]

**Q20. What other action or resources should accompany the implementation and communication of the CMO PA guidelines?**

[Prompts: CMO PA Guidelines communication strategy

A campaign with TV, radio, social media advertising,

Better resourcing to support the campaign

Inclusion of communication experts on different platforms

Coordinated approach with other health issues]

## Moving Medicine (MM)

**Q21. Do you know about MM? (yes/no)**

[MM is an online suite of resources that provide time specific consultations for HCP across 11 conditions]

*Skip to next section if unaware*

**Q22. Do you currently use MM resources? (yes/no)**

Q20a. If you do use it, how do you use it?

Q20b. If you don’t use it, why not?

**Q23. What works well and why?**

[Prompts: Content, coverage, access, style?]

**Q24. What does not work well and why?**

[Prompts: Content, coverage, access, style?]

**Q25. In your opinion what could be improved about Moving Medicine to make it more fit for your purpose as a clinician?**

# COVID-19

**Q26. Has COVID-19 changed the frequency or way you give PA advice? (yes/no)**

**Q27. If you gave PA advice during COVID-19 can you give an example of where, how and why you gave PA advice?**

**Q28. Did you give any specific advice to reduce sedentary behaviour during isolation periods?**

**Q29. Did you target any specific groups?**

**Q30. Can you give an example of where you have done this?**

**Q31. Is there anything else that you would like to add about delivering PA advice before we finish or anything you have not said?**

[Thank the participant and remind them of the contact details on the PIS should they have any questions, want to request a lay summary, etc.]

SAVE TRANSCRIPTION
